# Multi-Cohort Transcriptomic Integration Identifies Overlapping Immune and Antigen-Presentation Features in Alzheimer’s Disease and Hashimoto’s Thyroiditis

**DOI:** 10.3390/genes17101261

**Published:** 2026-10-11

**Authors:** Shuting Zhong, Guanfeng Xie, Zhaohui Zhang

**Affiliations:** Department of Neurology, Renmin Hospital of Wuhan University, Wuhan 430060, China

**Keywords:** Alzheimer’s disease, Hashimoto’s thyroiditis, transcriptomics, antigen presentation, MHC class II, neuroinflammation, thyroid autoimmunity

## Abstract

Background: Thyroid disorders and Alzheimer’s disease (AD) have been linked epidemiologically, but whether AD brain tissue and thyroid tissue affected by Hashimoto’s thyroiditis (HT) share molecular features remains unclear. Methods: Differential expression was estimated in AD (GSE122063) and HT (GSE138198) discovery cohorts using limma, with donor-level blocking and adjustment for age, sex and post-mortem interval where available. Shared genes were tested against chance within the genes measured in both cohorts, separately for each direction of change; characterized by enrichment analysis; related to marker-based cell-state scores; and evaluated in two AD cohorts, three thyroid cohorts including one with concurrently collected normal thyroid (GSE165724), and a single-nucleus AD dataset (GSE138852). Results: The discovery analyses identified 3593 AD- and 3806 HT-associated genes. The overlap was direction-specific: genes upregulated in both diseases exceeded chance (117 vs. 83.2 expected; *p* = 8.9 × 10^−5^), whereas the other direction combinations did not. Co-upregulated genes were enriched for MHC class II antigen presentation (FDR = 0.004), complement (FDR = 0.002), microglial activation (FDR = 0.017) and T-cell activation (FDR = 1.7 × 10^−4^), and recurred in two independent thyroid datasets and the within-study LT+ versus LT− comparison, with less consistent, region-dependent recurrence in AD cohorts. This shared component tracked immune cell-state signals—glial/myeloid activation in AD and lymphocytic infiltration in HT. Fourteen co-upregulated genes, including *ITGAX*, *CD22* and *BST2*, met prespecified recurrence and detectability criteria. Conclusions: AD brain tissue and thyroid tissue affected by HT exhibit a co-upregulated immune and antigen-presentation transcriptional program that reflects immune activation in both tissues. These overlapping features are associative and do not establish a causal relationship between AD and HT.

## 1. Introduction

Alzheimer’s disease (AD) is a progressive neurodegenerative disorder in which synaptic dysfunction, protein aggregation, glial activation, metabolic disturbance, and immune dysregulation interact across different disease stages [1,2]. Large genetic studies have implicated immune regulation, lipid metabolism, and endocytic pathways in AD susceptibility [3,4], and transcriptomic studies of human brain tissue have repeatedly identified downregulation of synaptic programs together with activation of immune- and glia-related programs [5,6,7]. Single-nucleus studies have shown that these changes are highly cell-type-dependent [8,9,10].

Hashimoto’s thyroiditis (HT) is an organ-specific autoimmune disorder characterized by lymphocytic infiltration, thyroid autoantibody and progressive loss of thyroid function [11,12,13]. Thyroid tissue from patients with HT shows prominent immune antigen-presentation signals [14]. HT, autoimmune hypothyroidism, hypothyroidism of any cause and variation in thyroid hormone levels are related but distinct entities. Most epidemiological evidence linking the thyroid to cognition concerns thyroid function rather than thyroid autoimmunity: a meta-analysis reported a higher prevalence of hypothyroidism among patients with AD while emphasizing uncertainty about temporal direction [15], genetic studies of thyroid-stimulating hormone and AD have yielded model-dependent results [16], and an early population-based study found no significant excess of AD among individuals with histologically confirmed HT [17]. Whether thyroid autoimmunity itself is related to AD therefore remains uncertain.

At the molecular level, a comparison of AD brain and HT thyroid tissue faces specific difficulties. Both tissues contain disease-associated immune cells—activated microglia in AD and infiltrating lymphocytes in HT—so shared signals may arise from immune cell content rather than from a shared disease mechanism. Large lists of differentially expressed genes can also intersect substantially by chance, and multi-region brain datasets contain repeated measurements from the same donors.

The aim of this study was to identify transcriptional features shared by AD brain tissue and HT thyroid tissue that exceed those expected by chance, to characterize their biological content and relation to immune cell states, and to evaluate their recurrence in independent cohorts.

## 2. Materials and Methods

### 2.1. Study Design and Public Datasets

**Figure 1:**
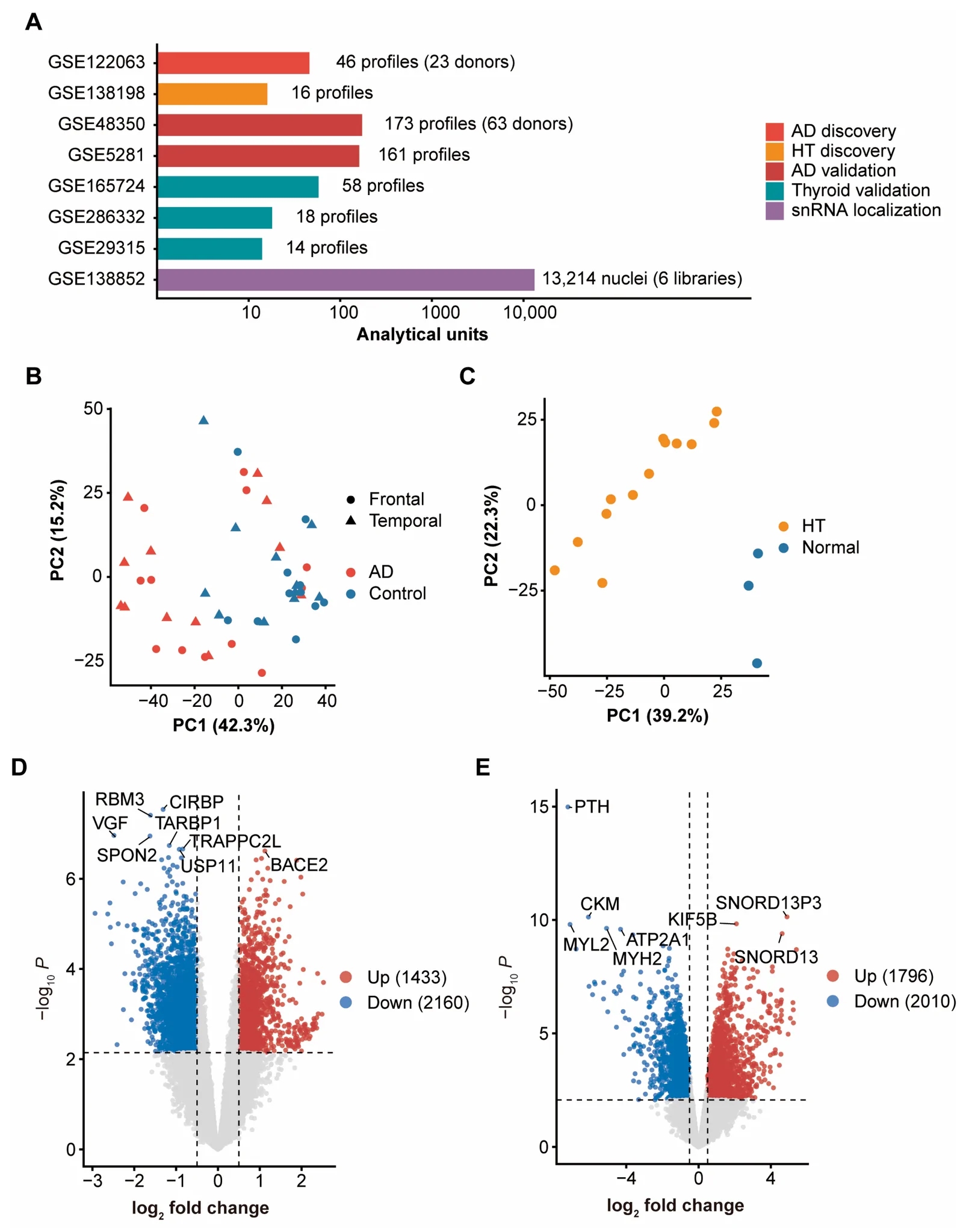
Cohort and discovery-cohort differential expression. (**A**) Datasets and analytical units, colored by role (log scale). (**B**,**C**) Principal component analysis of GSE122063 (AD; color, diagnosis; shape, region) and GSE138198 (HT). (**D**,**E**) Volcano plots for the donor-blocked, covariate-adjusted AD model and the HT model; dashed lines mark |log_2_FC| = 0.5 and FDR = 0.05. The eight genes with the smallest *p* values are labeled.

Eight publicly available human datasets from the NCBI Gene Expression Omnibus (GEO) were analyzed (Table 1; Figure 1A). GSE122063 and GSE138198 were the AD and HT discovery cohorts. GSE48350 and GSE5281 were used as independent AD cohorts. GSE165724, GSE286332 and GSE29315 were used as independent thyroid cohorts; GSE138852 was used to describe the cellular localization of candidate genes in the AD brain. The available demographic, pathological and technical characteristics of each dataset are summarized in Table 2 and Supplementary Table S7.

GSE122063 contains Agilent microarray profiles of the frontal and temporal cortex from 12 AD and 11 control donors, with technical replicates [5]. Technical replicates were averaged within donor and region, yielding 46 profiles from 23 donors; age, sex and post-mortem interval (PMI) were available and did not differ significantly between groups (Supplementary Table S1b). GSE138198 (Affymetrix Human Gene 1.0 ST) was used to compare 13 HT samples with three normal thyroid samples [14]. The three normal profiles were obtained from the array vendor’s data resource rather than being collected with the HT samples, so disease status and sample source are fully confounded in this comparison.

GSE48350 (Affymetrix Human Genome U133 Plus 2.0) contains profiles from four brain regions [6]. Because control donors spanned 20–99 years whereas AD donors were 60–95 years, controls younger than 60 years were excluded, leaving 173 profiles from 63 donors (35 control, 28 AD). GSE5281 contains 161 profiles of laser-captured neurons from six brain regions [18]; donor identifiers are not provided in GEO. GSE165724 contains RNA-seq profiles of normal thyroid obtained during laryngeal-cancer surgery (n = 12) and of histologically normal thyroid adjacent to papillary thyroid carcinoma (PTC) with (LT+, n = 21) or without (LT−, n = 25) lymphocytic thyroiditis, all collected in the same study [19]. GSE286332 compares HT with non-HT thyroid within a PTC background (9 vs. 9), and GSE29315 compares HT with thyroid hyperplasia (6 vs. 8). None of the thyroid validation cohorts is a conventional HT-versus-healthy comparison; GSE165724 was included because it provides normal thyroid and lymphocytic-thyroiditis tissue collected within one study. GSE138852 contains 13,214 entorhinal-cortex nuclei from six AD and six control donors, sequenced as six libraries that each pooled two donors of the same diagnosis [8].

### 2.2. Preprocessing

The input scale, transformation, gene-level summarization and quality control of each dataset are listed in Supplementary Table S2. For microarray series matrices, the scale of each matrix was verified individually rather than inferred from a generic rule. GSE122063, GSE138198 and GSE29315 were supplied on a log_2_ scale; GSE5281 was log_2_(x + 1)-transformed; and GSE48350, which is supplied on a linear MAS5-scaled scale, was log_2_-transformed. When several probes mapped to one gene symbol, the probe with the highest median expression was retained. For the two RNA-seq cohorts (GSE286332 and GSE165724), raw read counts were filtered with filterByExpr, normalized by the trimmed mean of M-values method and analyzed with voom [20]. Principal component analysis, sample-level expression distributions, and top-genes heatmaps were used for quality assessment; no sample was excluded. Expression values and effect sizes were not pooled across platforms.

### 2.3. Differential Expression

Differential expression was estimated with limma [21]. In GSE122063, the combined model was ~group + region + age + sex + PMI, and within-donor correlation between the two regions was modeled with duplicateCorrelation, using donor as the blocking factor [22]. A model with donor blocking but without covariates was fitted as a sensitivity analysis. Region-specific analyses (one profile per donor) used ~group + age + sex + PMI. In GSE48350 the combined model was ~group + region + age + sex with donor blocking, and region-specific models were ~group + age + sex. In GSE5281, donor identity could not be recovered, so the combined model (~group + region + age + sex) treats profiles as independent; region-specific analyses, in which each donor contributes one profile, were therefore used for all recurrence counts. GSE138198, GSE286332 and GSE29315 were analyzed with ~group because no covariates were available. GSE165724 was analyzed with ~group + age + sex for LT+ versus normal thyroid and for LT+ versus LT− tissue. *p* values were adjusted with the Benjamini–Hochberg method, and genes with FDR < 0.05 and |log_2_ fold change| > 0.5 were considered differentially expressed (DEGs).

### 2.4. Overlap Testing and Enrichment

The analysis universe was defined as the 18,387 genes tested in both discovery cohorts. The number of genes significant in both cohorts was compared with the number expected under independence using the hypergeometric distribution and a permutation test in which AD DEG sets of the observed size were drawn at random from the universe 10,000 times. Because direction matters for interpretation, overlaps were also tested separately for each of the four combinations of AD and HT direction (up/up, down/down, AD down/HT up, AD up/HT down). The genome-wide agreement of the two disease effects was summarized as the Spearman correlation of the moderated t statistics.

Gene Ontology biological-process and KEGG over-representation analyses were performed with clusterProfiler [23], with the universe argument set explicitly to the Entrez identifiers of the 18,387 common genes, for all shared genes and separately for concordant, discordant and co-upregulated genes. Enrichment was interpreted as over-representation of annotated genes, not as pathway activation.

### 2.5. Relation to Cell-State Signals

Marker-based cell-state scores were calculated as the mean standardized expression of predefined marker sets for 15 immune, glial, neuronal and vascular cell states (Supplementary Table S9). They summarize coordinated marker expression and are not estimates of cell proportions. To determine whether the shared component was linked to immune cell states, the AD discovery model was refitted with microglia/macrophage and astrocyte scores as additional covariates, and the HT model with T-cell and B-cell scores, and the overlap tests were repeated with the adjusted AD model, the adjusted HT model and both. Because three microglial marker genes (*TYROBP*, *C1QA*, *C1QB*) were themselves co-upregulated, the adjustment was repeated with scores recomputed after excluding these genes.

### 2.6. Candidate Prioritization

Candidate genes were selected from the co-upregulated component by a fixed rule without manual or literature-based input. A gene was prioritized if it (C1) was a DEG in both discovery cohorts with increased expression in both; (C2) showed FDR < 0.05 in the same direction in at least one independent AD analysis (GSE48350 combined or any region-specific analysis in GSE48350 or GSE5281); (C3) showed FDR < 0.05 in the same direction in at least one independent thyroid analysis (GSE165724 LT+ vs. normal, GSE286332 or GSE29315); and (C4) was detected in at least 10% of nuclei of at least one major cell type in GSE138852. Genes meeting all criteria were ranked by the number of supporting AD and thyroid analyses and then by the sum of their discovery *p*-value ranks. The complete ranked list of all shared genes, with each criterion, is provided in Supplementary Table S5, and the status of the nine genes highlighted in the initial version of this analysis is shown in Supplementary Table S5b. Publication counts were not used.

### 2.7. Co-Expression Analysis

Weighted gene co-expression network analysis (WGCNA) [24] was performed in each discovery cohort using the 3000 most variable genes, a soft-thresholding power selected as the first power reaching a scale-free fit R^2^ ≥ 0.85, biweight midcorrelation, an unsigned topological-overlap matrix, a minimum module size of 30 and a merge height of 0.25. In GSE122063, module eigengenes were averaged within donor before correlation with disease status (n = 23 donors).

### 2.8. Single-Nucleus Localization

Processed counts and cell annotations from GSE138852 were used [8]. Processing followed established recommendations for single-cell data [25]. Expression was normalized as log1p counts per 10,000, and uniform manifold approximation and projection were computed from the first 20 principal components of 1200 variable genes. Because each library pooled two donors and donor identity cannot be recovered from the deposited data, disease comparisons were not performed; individual nuclei were not treated as independent replicates [26], and the analysis was limited to describing candidate expression (mean normalized expression and fraction of nuclei with detected expression) across cell types.

### 2.9. Exploratory Mendelian Randomization

A bidirectional two-sample Mendelian randomization (MR) screen based on FinnGen summary statistics [27] is reported in the Supplementary Material (Supplementary Figure S1; Supplementary Table S8) as an exploratory analysis. Instruments were selected at *p* < 5 × 10^−8^ or, in an expanded screen, *p* < 1 × 10^−6^ with F ≥ 10 and a minimum inter-variant distance of 10 Mb; inverse-variance weighting was the principal estimator [28,29]. Benjamini–Hochberg and Bonferroni corrections were applied across all 27 inverse-variance-weighted models. Distance-based filtering is not equivalent to reference-panel linkage-disequilibrium clumping, exposure and outcome were both drawn from FinnGen, and variant retrieval was incomplete; the results were therefore not interpreted causally [30].

### 2.10. Statistical Analysis and Reproducibility

All analyses were performed in R. Tests were two-sided. The revised analysis scripts (differential expression, overlap and candidate selection, supporting analyses) are provided as Supplementary Code S1, together with R package versions.

## 3. Results

### 3.1. Discovery Cohorts

Principal component analysis separated HT from normal thyroid more clearly than AD from control brain (Figure 1B,C). In GSE122063, the two regional profiles from the same donor were strongly correlated (consensus within-donor correlation 0.62). With donor blocking and adjustment for age, sex and PMI, 3593 of 32,080 genes were differentially expressed (1433 up, 2160 down; Figure 1D); donor blocking without covariates gave 3706 DEGs. In GSE138198, 3806 of 23,307 genes were differentially expressed (1796 up, 2010 down), led by *PTH*, *CKM* and *MYL2* (Figure 1E). These leading signals are compatible with differences in tissue composition or sampling between the HT samples and the vendor-derived normal samples, and the HT discovery contrast should be regarded as hypothesis-generating.

### 3.2. Co-Expression and Cell-State Patterns

**Figure 2:**
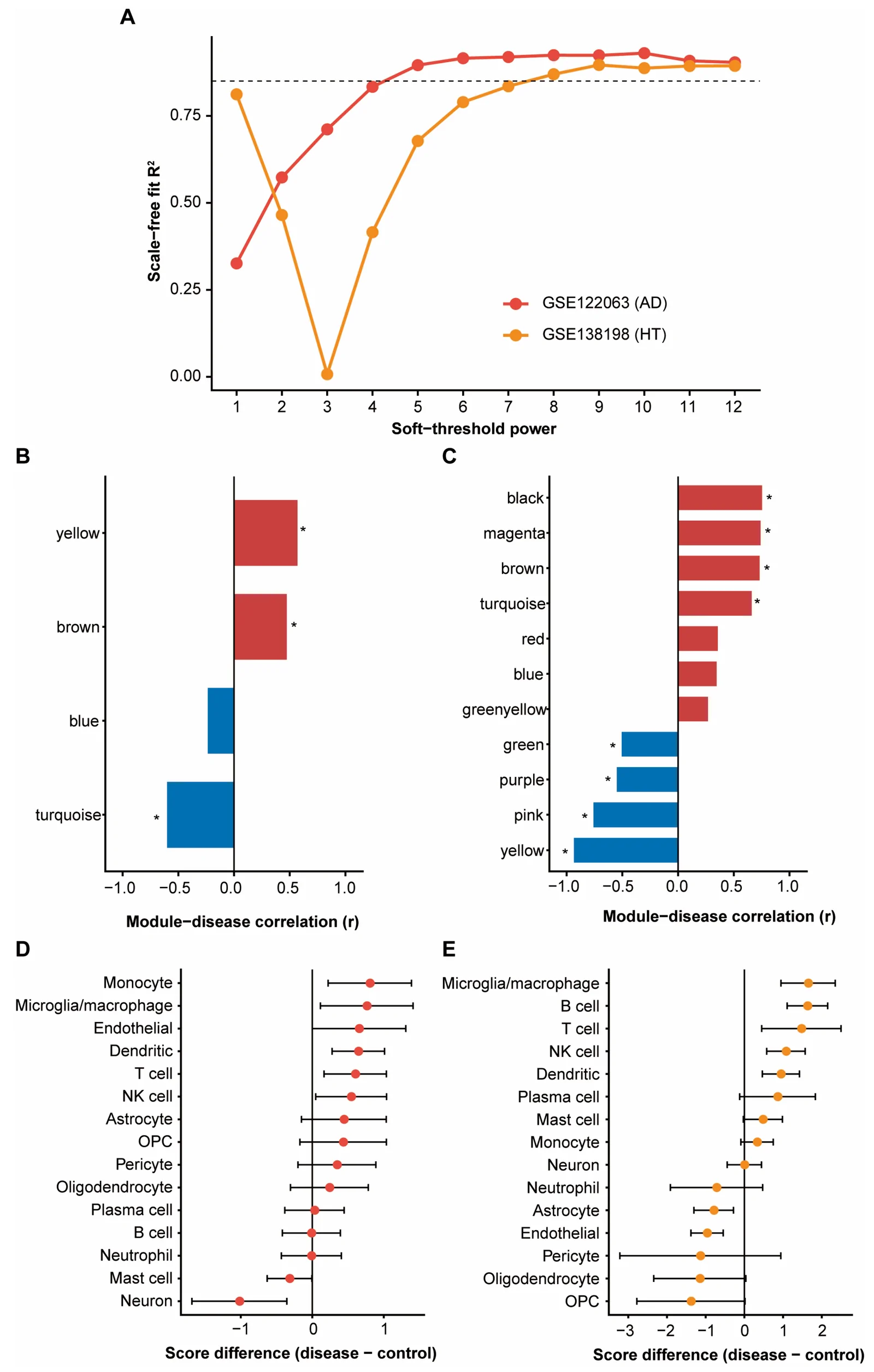
Co-expression and marker-based cell-state patterns. (**A**) Scale-free topology fits by soft-threshold powers. (**B**) Donor-level module–disease correlations in GSE122063 (n = 23 donors). * *p* < 0.05. (**C**) Module–disease correlations in GSE138198. * *p* < 0.05. (**D**,**E**) Differences in marker-based cell-state scores between disease and control (mean and 95% CI) in GSE122063 (donor level) and GSE138198. Scores are standardized marker-expression summaries, not cell proportions.

At the donor level, three AD modules were associated with disease status (MEturquoise *r* = −0.60, *p* = 0.0024; MEyellow *r* = 0.57, *p* = 0.0045; MEbrown *r* = 0.47, *p* = 0.023; Figure 2A,B). In HT, MEyellow showed the strongest association (*r* = −0.93, *p* = 1.2 × 10^−7^), with several further modules at |r| ≥ 0.5 (Figure 2C); these correlations are based on three control samples. Marker-based scores indicated higher microglia/macrophage, monocyte, dendritic and T-cell scores and a lower neuronal score in AD donors (Figure 2D), and markedly higher B-cell, T-cell, NK-cell and microglia/macrophage (myeloid) scores in HT (Figure 2E; Supplementary Table S9). Both discovery contrasts therefore carry a substantial immune cell-state component.

### 3.3. AD and HT Shared a Co-Upregulated Immune and Antigen-Presentation Component

**Figure 3:**
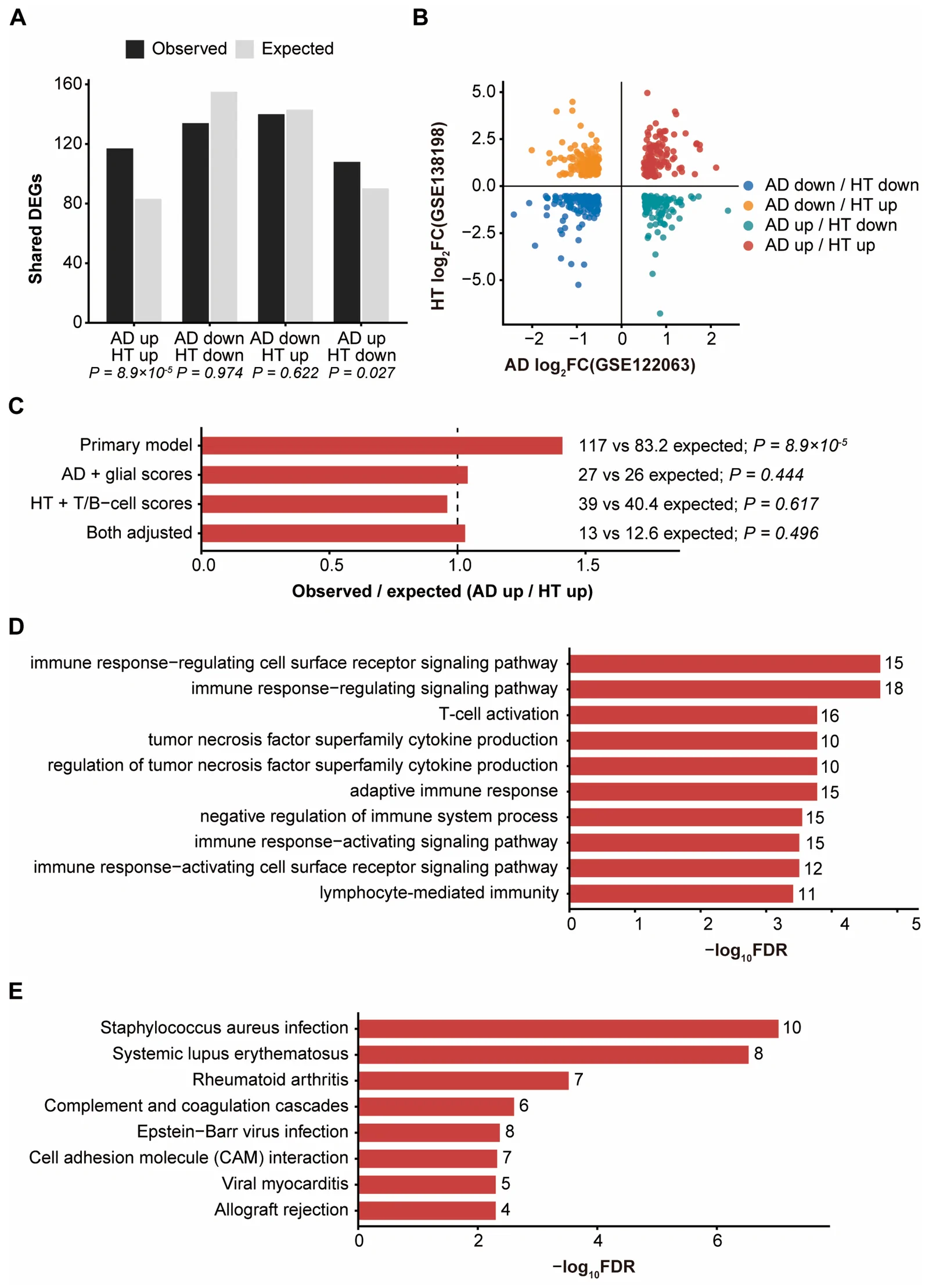
Direction-specific overlap and immune features of co-upregulated genes. (**A**) Observed and expected numbers of shared DEGs in each direction combination within the 18,387 genes tested in both cohorts (hypergeometric *p* values). (**B**) Discovery log_2_ fold changes of the 499 shared genes. (**C**) Observed/expected ratio of co-upregulated genes in the primary models and after adjustment for cell-state scores. (**D**,**E**) Top GO biological-process and KEGG terms (by FDR) for the 117 co-upregulated genes using the common-gene universe; numbers are gene counts. MHC class II antigen-presentation, microglial-activation and complement terms are listed in Supplementary Table S4.

Within the 18,387 genes tested in both cohorts, 499 genes were differentially expressed in both discovery cohorts (251 concordant, 248 discordant). Because both gene lists are large, the overlap was tested against chance. The overlap was direction-specific: genes upregulated in both diseases were clearly over-represented (117 observed vs. 83.2 expected; fold enrichment 1.41; hypergeometric *p* = 8.9 × 10^−5^), with a weaker excess of AD-up/HT-down genes (108 vs. 90.2; *p* = 0.027), whereas co-downregulated and AD-down/HT-up genes were not over-represented (*p* = 0.97 and 0.62; Figure 3A,B). Taken together across all directions, the 499 shared genes did not exceed the number expected from the sizes of the two lists (expected 471.5; *p* = 0.070; permutation *p* = 0.069), and the genome-wide correlation of AD and HT t statistics was close to zero (Spearman ρ = 0.012). The co-upregulated excess was robust to the AD model specification, being present with donor blocking alone (*p* = 1.4 × 10^−3^) and without donor blocking (*p* = 2.8 × 10^−4^; Supplementary Table S3). The unadjusted cross-tissue overlap is therefore concentrated in a specific co-upregulated component rather than in the intersection as a whole.

The co-upregulated genes were enriched for immune and antigen-presentation programs (Figure 3D,E; Supplementary Table S4), including immune response-regulating signaling (FDR = 1.8 × 10^−5^), T-cell activation and adaptive immune response (both FDR = 1.7 × 10^−4^), antigen processing and presentation of exogenous peptide antigen via MHC class II (FDR = 0.004), MHC class II protein complex assembly (FDR = 0.012), microglial cell activation (FDR = 0.017), complement and coagulation cascades (KEGG; FDR = 0.002) and antigen processing and presentation (KEGG; FDR = 0.023). The genes driving these terms included *HLA-DRB1*, *HLA-DRB5*, *HLA-DQA1*, *IFI30*, *CD86*, *C1QA*, *C1QB*, *C1QC*, *C3AR1*, *C5AR1*, *TYROBP*, *FCGR2A* and *PTPRC*—genes characteristic of activated myeloid and antigen-presenting cells. Discordant genes showed no enriched term, and the 499 shared genes as a whole showed only weak enrichment (e.g., positive regulation of leukocyte activation, FDR = 0.012). Disease-labeled KEGG categories (e.g., Staphylococcus aureus infection, systemic lupus erythematosus) reflect shared complement and HLA annotation and do not imply similarity to the named diseases.

### 3.4. The Shared Immune Component Tracks Immune Cell-State Signals

Both discovery contrasts carried strong immune cell-state signals (Figure 2D,E). Adjusting the AD model for microglia/macrophage and astrocyte scores reduced the co-upregulated overlap to the level expected by chance (27 vs. 26.0 expected; *p* = 0.44), as did adjusting the HT model for T- and B-cell scores (39 vs. 40.4; *p* = 0.62) or both (13 vs. 12.6; *p* = 0.50; Figure 3C; Supplementary Table S3). The same result was obtained when the scores were recomputed without the three microglial markers that were themselves co-upregulated (AD-adjusted *p* = 0.17; both adjusted *p* = 0.64; Supplementary Table S3). The shared immune and antigen-presentation component is therefore closely tied to glial/myeloid activation in AD and lymphocytic infiltration in HT, indicating that the overlapping features are expressed by, or co-vary with, activated immune cell populations in both tissues.

### 3.5. Independent Cohorts

**Figure 4:**
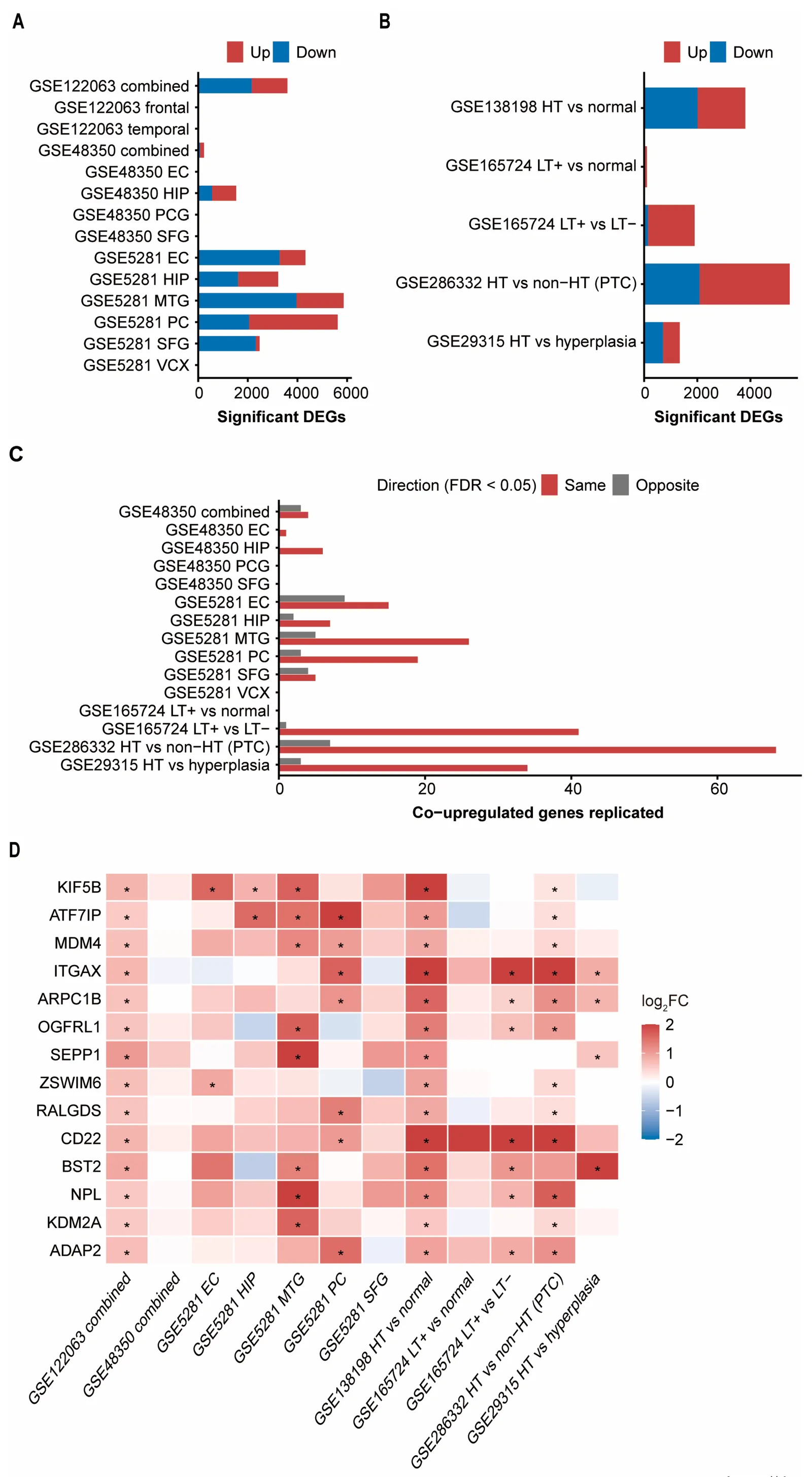
Independent cohorts and rule-based candidates. (**A**,**B**) Numbers of DEGs in AD and thyroid analyses. EC, entorhinal cortex; HIP, hippocampus; PCG, postcentral gyrus; SFG, superior frontal gyrus; MTG, middle temporal gyrus; PC, posterior cingulate; VCX, primary visual cortex. (**C**) Co-upregulated genes reaching FDR < 0.05 in the same or opposite direction in each independent analysis. (**D**) log_2_ fold changes of the 14 prioritized genes across discovery and independent analyses (truncated at ±2); * FDR < 0.05; blank cells, gene not measured.

After log_2_ transformation and age matching, GSE48350 yielded 235 DEGs in the donor-blocked combined analysis (within-donor correlation 0.46) and 20–1532 DEGs in region-specific analyses. GSE5281 region-specific analyses yielded 2473–5861 DEGs in five regions and one DEG in primary visual cortex (Figure 4A). Of the 478 testable shared genes, 46 replicated in the same direction in the GSE48350 combined analysis and 75–201 in five of the six GSE5281 regions (none in primary visual cortex; Supplementary Table S6).

In the thyroid cohorts, 141 of 417 testable shared genes replicated in the same direction in GSE286332 (47 in the opposite direction) and 57 of 285 in GSE29315 (22 opposite). In GSE165724, only 108 genes differed between LT+ tissue and normal thyroid, and none of the shared genes replicated in this comparison (Figure 4B,C). This contrast provided limited independent disease information after covariate adjustment because the normal thyroid donors were all male and older (60.3 ± 9.2 years), whereas the LT+ donors were predominantly female (19 of 21) and younger (40.4 ± 13.4 years) (Table 2). Within the PTC-adjacent tissue in the same study, where these factors are more balanced, 41 of 106 testable co-upregulated genes were increased in LT+ relative to LT− tissue (one decreased). Co-upregulated genes also replicated in 68 of 106 testable genes in GSE286332 and 34 of 67 in GSE29315, but in only 0–26 genes per AD validation analysis (Figure 4C).

### 3.6. Rule-Based Candidates

Of the 117 co-upregulated genes, 47 met the AD recurrence criterion, 73 the thyroid recurrence criterion and 46 the single-nucleus detection criterion; 14 met all criteria (Table 3; Figure 4D; Supplementary Table S5): *KIF5B*, *ATF7IP*, *MDM4*, *ITGAX*, *ARPC1B*, *OGFRL1*, *SEPP1* (*SELENOP*), *ZSWIM6*, *RALGDS*, *CD22*, *BST2*, *NPL*, *KDM2A* and *ADAP2*. Several are expressed by immune cells (*ITGAX*, *CD22*, *BST2*, *ARPC1B*, *ADAP2*), in keeping with the immune character of the shared component. The nine genes highlighted in the initial version of this analysis (*CIRBP*, *IDS*, *CAMLG*, *LETMD1*, *RBM3*, *RHOJ*, *BDH1*, *CAMKK2* and *CD44*) all remained differentially expressed in both discovery cohorts; their recurrence profiles are reported in Supplementary Table S5b. Seven of them change in opposite directions in AD and HT, a pattern that was not over-represented beyond chance, and none met all prespecified criteria.

### 3.7. Cellular Localization in the AD Brain

**Figure 5:**
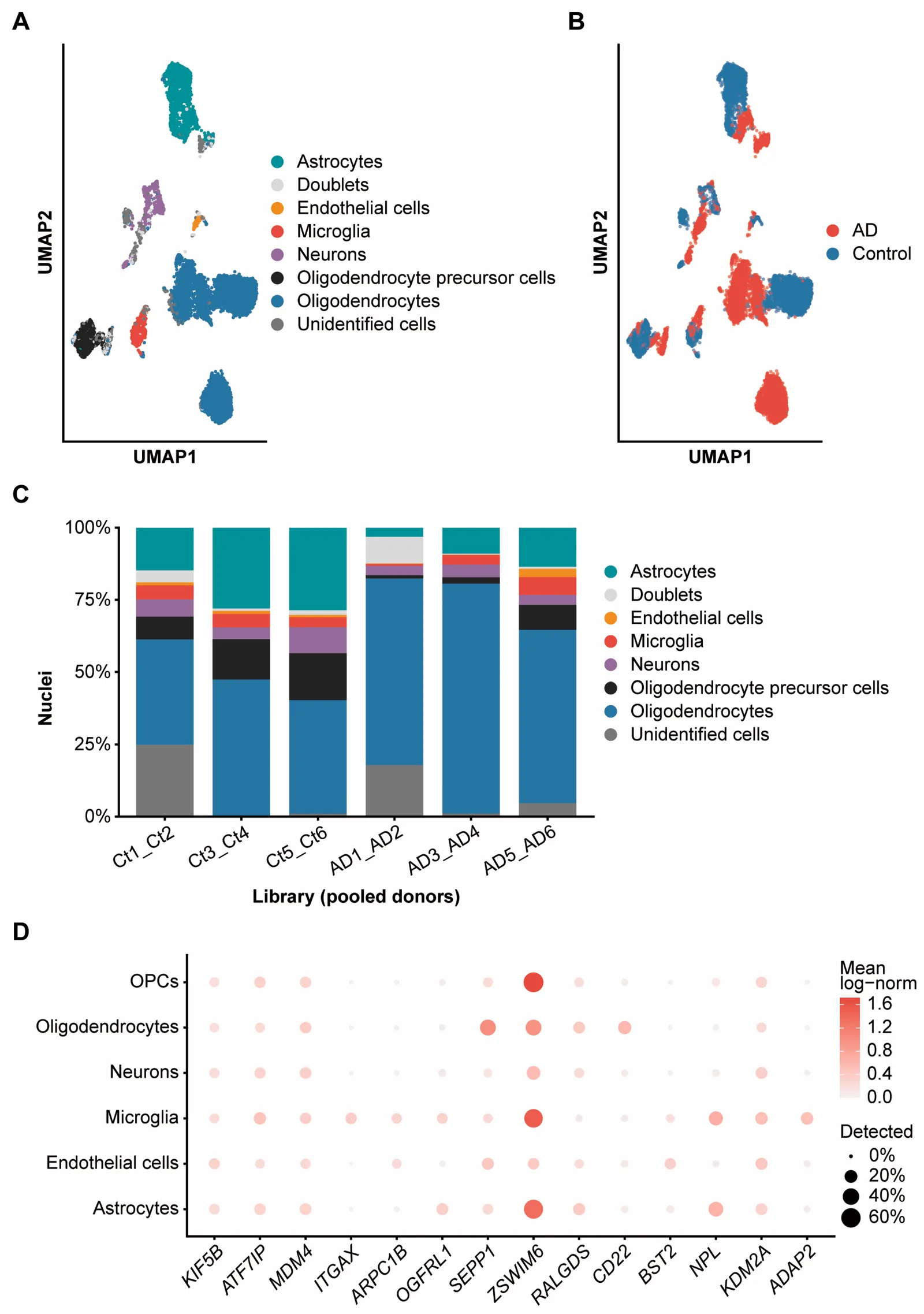
Cellular localization of candidates in the AD entorhinal cortex (GSE138852). (**A**,**B**) UMAP colored by annotated cell type and by diagnosis. (**C**) Cell-type composition of each library; each library pools two donors of the same diagnosis. (**D**) Fraction of nuclei with detected expression (size) and mean log-normalized expression (color) of the 14 candidates across major cell types. The data are descriptive; no disease comparison was performed.

GSE138852 comprised 13,214 nuclei (6673 AD, 6541 control) from six pooled libraries, with marked library-to-library variation in cell-type composition (Figure 5A–C). *ITGAX*, *ARPC1B*, *ATF7IP*, *KDM2A* and *ADAP2* were detected most frequently in microglia, *SEPP1* and *CD22* in oligodendrocytes, *NPL*, *MDM4*, *OGFRL1* and *RALGDS* in astrocytes, and *KIF5B* and *BST2* in endothelial cells; *ZSWIM6* was broadly detected (Figure 5D). These data describe where candidate transcripts are detected and do not test disease effects.

### 3.8. Exploratory Mendelian Randomizations

The MR screen is reported in Supplementary Figure S1 and Supplementary Table S8. Of 27 inverse-variance-weighted models, 24 relied on one or two variants. The inverse association between genetically predicted AD and strict autoimmune hypothyroidism after exclusion of the APOE region (two variants; odds ratio 0.795, 95% CI 0.703–0.899; FDR = 0.0012) was not supported by the corresponding three-variant full model (odds ratio 1.02, *p* = 0.195), which showed heterogeneity (Cochran’s Q *p* = 3.2 × 10^−4^). Autoimmune hypothyroidism is a clinically defined phenotype that overlaps with, but is not equivalent to, HT. These results provide no evidence for a causal relationship in either direction.

## 4. Discussion

This multi-cohort analysis identified overlapping immune and antigen-presentation features in AD brain tissue and HT thyroid tissue. The overlap was direction-specific: genes upregulated in both diseases exceeded chance, were enriched for MHC class II antigen presentation, complement, microglial activation and T-cell activation, and recurred in two independent thyroid datasets and the within-study LT+ versus LT− comparison, with region-dependent recurrence in AD cohorts. Other direction combinations, and the intersection as a whole, did not exceed chance, indicating that the shared biology is concentrated in this co-upregulated immune program rather than spread across the transcriptome.

The content of the shared program is consistent with current understanding of both diseases. In AD, MHC class II-positive microglia accumulate around lesions [31], microglial antigen-presentation states are linked to genetic risk and disease progression [10,32], and complement and TYROBP-dependent microglial activation are part of the neuroinflammatory response [1,2]. In HT, HLA class II-positive antigen-presenting cells and infiltrating lymphocytes are defining features of thyroid autoimmunity [11,12,13,33]. The close relation between the shared component and cell-state scores indicates that these overlapping features are carried by activated immune cell populations—glial and myeloid cells in AD, lymphocytes and antigen-presenting cells in HT. The two diseases therefore share an immune activation and antigen-presentation program at the tissue level, although the cell types that express it differ between brain and thyroid. Whether this reflects a common upstream driver, such as systemic immune activation, or parallel tissue responses cannot be determined from transcriptomic data.

The HT discovery contrast remains the weakest element. Its three normal samples came from a different source, and its leading genes (*PTH*, *CKM*, *MYL2*) point to differences in tissue sampling. We therefore added GSE165724, in which normal and thyroiditis tissue were collected within one study. The direct LT+ versus normal contrast yielded no replication and was difficult to interpret because sex and age were almost completely separated between groups, whereas the within-study LT+ versus LT− comparison reproduced a substantial part of the co-upregulated component. Recurrence in GSE286332 and GSE29315, which also use within-study comparators, points the same way. These analyses support the immune component but do not constitute replication of an HT-versus-healthy signature, and the thyroid findings should be regarded as hypothesis-generating.

Recurrence in the independent AD cohorts varied by region and dataset. It was most frequent in the GSE5281 laser-captured neuronal profiles and in GSE48350 hippocampus, and absent in primary visual cortex, a region involved late in AD. GSE5281 does not provide donor identifiers, so its combined analysis cannot account for within-donor correlation; for this reason, only its region-specific analyses were used to count recurrence. Covariates such as Braak stage, RNA integrity and array batch were unavailable for most cohorts (Table 2).

The single-nucleus data were used only to describe where candidate transcripts are detected. Each library pooled two donors, so donor-resolved pseudobulk analysis is not possible from the deposited data, and with three libraries per group any library-level comparison would be severely underpowered. The expression of several candidates in microglia is consistent with the bulk-tissue interpretation. Curated ligand–receptor scoring was not included because expression-based communication scores largely reflect baseline expression and the number of edges per gene, and depend strongly on the method and resource chosen [34,35]. The MR screen was moved to the Supplementary Material because it rests on sparse instruments, distance-based filtering, incomplete variant retrieval and a single data source for exposure and outcome.

This study has several limitations. The HT discovery contrast is confounded by sample source. Most datasets lack information on disease stage, RNA quality and batch, and thyroid function or autoantibody status is unavailable for the brain donors. Marker-based scores are proxies for cell composition; bulk data cannot separate changes in immune cell abundance from changes in activation state within those cells, and cell-type-resolved data from both tissues would be needed to do so. Only the co-upregulated component exceeded chance, so other shared genes, including those changing in opposite directions, should not be interpreted as shared disease features. Tissue-level transcriptomics cannot establish whether any candidate is upstream of disease, and the findings do not establish a causal relationship between thyroid autoimmunity and AD, which would require prospective cohorts, donor-matched thyroid and brain phenotyping, and appropriately powered genetic analyses.

## 5. Conclusions

Multi-cohort analysis identified overlapping AD- and HT-associated transcriptomic features in the form of a co-upregulated immune program involving MHC class II antigen presentation, complement, microglial activation and T-cell activation. In the primary models, this program exceeded chance, recurred in two independent thyroid datasets and the within-study LT+ versus LT− comparison, showed region-dependent recurrence in AD cohorts, and tracked activated immune cell states in both tissues. Fourteen rule-based candidates, including *ITGAX*, *CD22* and *BST2*, warrant cell-type-resolved and experimental evaluation. The findings are associative and do not establish diagnostic value or a causal relationship between AD and HT.

## Figures and Tables

**Table 1:** Datasets, analytical roles and models.

Dataset	Tissue/Comparison	Platform	Analytical Units	Model	Role
GSE122063	Frontal and temporal cortex; AD vs. control	Agilent SurePrint G3 Human GE v2 8 × 60K Agilent G3 8 × 60K	46 profiles (24 AD, 22 control) 46 profiles, 23 donors (12 AD, 11 control)	~group + region + age + sex + PMI; donor block	AD discovery
GSE138198	Thyroid; HT vs. normal (vendor-derived)	Affymetrix HuGene 1.0 ST	16 (13 HT, 3 normal)	~group	HT discovery
GSE48350	Four brain regions; AD vs. control ≥ 60 y	Affymetrix U133 Plus 2.0	173 profiles, 63 donors	~group + region + age + sex; donor block	AD validation
GSE5281	Six regions, laser-captured neurons	Affymetrix U133 Plus 2.0	161 profiles (donor IDs unavailable)	region-specific ~group + age + sex	AD validation
GSE165724	Normal thyroid; PTC-adjacent LT+/LT−	Illumina RNA-seq	58 (12 normal, 21 LT+, 25 LT−)	~group + age + sex (voom)	Thyroid validation
GSE286332	Thyroid; HT vs. non-HT, PTC background	Illumina RNA-seq	18 (9 vs. 9)	~group (voom)	Thyroid validation
GSE29315	Thyroid; HT vs. hyperplasia	Affymetrix U95Av2	14 (6 vs. 8)	~group	Thyroid validation
GSE138852	Entorhinal cortex snRNA-seq	10x Genomics	13,214 nuclei, 6 libraries (2 donors each)	descriptive	Cellular localization

**Table 2:** Available characteristics of the bulk datasets (mean ± SD; female/male counted per donor where donor identity is available, otherwise per profile; NA, not provided).

Dataset	Group	n	Age, Years	Female/Male	Other Available Information
GSE122063	AD	12 donors	80.9 ± 7.2	9/3	PMI 8.6 ± 4.0 h; Braak, RIN, batch NA
	Control	11 donors	78.8 ± 8.6	6/5	PMI 8.9 ± 3.1 h
GSE138198	HT	13	NA	12/1	thyroid function, antibodies NA
	Normal	3	NA	NA	vendor-derived
GSE48350	AD	28 donors (80 profiles)	85.4 ± 7.4	47/33 profiles	Braak III–VI in 72 of 80 profiles; PMI NA
	Control (≥60 y)	35 donors (93 profiles)	81.3 ± 10.1	48/45 profiles	PMI NA
GSE5281	AD	87 profiles	79.8 ± 6.9	37/50 profiles	donor IDs, PMI, Braak NA
	Control	74 profiles	79.5 ± 8.9	21/53 profiles	
GSE165724	LT+	21	40.4 ± 13.4	19/2	PTC-adjacent
	LT−	25	48.1 ± 17.4	19/6	PTC-adjacent
	Normal	12	60.3 ± 9.2	0/12	laryngeal-cancer thyroidectomy
GSE286332	HT/non-HT	9/9	NA	NA	PTC background
GSE29315	HT/hyperplasia	6/8	NA	NA	—

**Table 3:** Rule-based candidates from the co-upregulated component. AD values are from GSE122063 (donor-blocked, covariate-adjusted) and HT values are from GSE138198. Replication counts are analyses with FDR < 0.05 in the same direction.

Gene	AD log_2_FC	AD FDR	HT log_2_FC	HT FDR	AD Analyses Replicated	Thyroid Analyses Replicated	Cell Type with Highest Detection (Fraction)
KIF5B	0.77	0.0036	2.09	7.4 × 10^−7^	3	1	Endothelial (0.13)
ATF7IP	0.54	0.026	1.04	0.0021	3	1	Microglia (0.18)
MDM4	0.65	0.0040	0.90	0.026	2	1	Astrocytes (0.16)
ITGAX	0.76	0.015	2.11	0.013	1	2	Microglia (0.15)
ARPC1B	0.63	0.040	1.61	0.0061	1	2	Microglia (0.11)
OGFRL1	0.61	0.012	1.37	0.0077	1	1	Astrocytes (0.17)
SEPP1	1.05	0.0087	1.10	0.014	1	1	Oligodendrocytes (0.37)
ZSWIM6	0.66	0.019	0.97	0.0027	1	1	OPCs (0.65)
RALGDS	0.61	0.014	0.90	0.025	1	1	Astrocytes (0.18)
CD22	0.77	0.025	3.34	0.012	1	1	Oligodendrocytes (0.22)
BST2	0.91	0.0099	1.51	0.042	1	1	Endothelial (0.14)
NPL	0.56	0.018	1.20	0.029	1	1	Astrocytes (0.30)
KDM2A	0.55	0.023	0.57	0.026	1	1	Microglia (0.20)
ADAP2	0.67	0.031	0.96	0.021	1	1	Microglia (0.19)

## Data Availability

The expression datasets analyzed in this study are publicly available from the NCBI Gene Expression Omnibus under accession numbers GSE122063, GSE138198, GSE48350, GSE5281, GSE286332, GSE29315, and GSE138852. FinnGen summary statistics were obtained through the publicly accessible FinnGen results interface. Processed source-data tables underlying the custom manuscript panels accompany the manuscript.

## Supplementary Materials

The following supporting information can be downloaded at https://www.mdpi.com/article/10.3390/genes17101261/s1: Supplementary Code S1. The R script for bioinformatics analysis of the article. Supplementary Figure S1. Exploratory bidirectional Mendelian randomization screen based on FinnGen summary statistics. Supplementary Tables S1–S9: S1, differential-expression models and DEG counts (S1b, GSE48350 age matching and GSE122063 covariate balance); S2, dataset-specific preprocessing; S3, overlap tests; S4, enrichment results; S5, complete ranked list of shared genes (S5b, status of the nine originally prioritized genes); S6, replication in independent cohorts; S7, dataset characteristics; S8, MR models with multiple-testing correction; S9, cell-state score differences and donor-level WGCNA results.
